# Selection in spatial stochastic models of cancer: Migration as a key modulator of fitness

**DOI:** 10.1186/1745-6150-5-21

**Published:** 2010-04-20

**Authors:** Craig J Thalhauser, John S Lowengrub, Dwayne Stupack, Natalia L Komarova

**Affiliations:** 1 Department of Mathematics, University of California Irvine, Irvine, California, USA; 2 Department of Pathology, University of California San Diego, San Diego, California, USA

## Abstract

**Background:**

We study the selection dynamics in a heterogeneous spatial colony of cells. We use two spatial generalizations of the Moran process, which include cell divisions, death and migration. In the first model, migration is included explicitly as movement to a proximal location. In the second, migration is implicit, through the varied ability of cell types to place their offspring a distance away, in response to another cell's death.

**Results:**

In both models, we find that migration has a direct positive impact on the ability of a single mutant cell to invade a pre-existing colony. Thus, a decrease in the growth potential can be compensated by an increase in cell migration. We further find that the neutral ridges (the set of all types with the invasion probability equal to that of the host cells) remain invariant under the increase of system size (for large system sizes), thus making the invasion probability a universal characteristic of the cells selection status. We find that repeated instances of large scale cell-death, such as might arise during therapeutic intervention or host response, strongly select for the migratory phenotype.

**Conclusions:**

These models can help explain the many examples in the biological literature, where genes involved in cell's migratory and invasive machinery are also associated with increased cellular fitness, even though there is no known direct effect of these genes on the cellular reproduction. The models can also help to explain how chemotherapy may provide a selection mechanism for highly invasive phenotypes.

**Reviewers:**

This article was reviewed by Marek Kimmel and Glenn Webb.

## Background

Cancerous cells within a tumor compete with one another in a fast paced evolutionary system. At the molecular level, mutations are introduced into the tumoral genome; these mutations may be caused by inherited deficiencies, loss of mismatch repair systems, downregulation of the proofreading checkpoints, and chromosomal instabilities. At the cellular level, these mutations introduce changes in phenotype, some profound but many others subtle. It is the emergence of these mutations, as well as epigenetic events, which generates the incredible flexibility and adaptability of the cancer disease state.

While the effect of certain mutations on the cell's phenotype is reasonably well understood (induction of K-Ras, loss of p53 and/or Rb, overexpression of matrix metalloproteases [1]), it is conceptually difficult to quantify and analyze the level of genetic heterogeneity within a given tumor. It is even more difficult to measure the forces of natural selection in anything other than broad, descriptive terms.

Speaking in broad evolutionary terms, we would like to understand what cellular characteristics make certain cells more fit than others. If a mutant is introduced in a cell colony, what combinations of the mutant characteristics and "background" characteristics make the mutant cells win the evolutionary competition?

The idea that cancer is an evolutionary process has been applied successfully by many computational biologists, as it allows them to use methods of theoretical population biology and ecology [2-8]. Here we focus on two types of phenotypic changes induced by mutations. The first type involves mutations in genes affecting cell proliferation. Activation of some oncogenes, or inactivation of tumor suppressor genes, change the cells' reproductive capacity, and are thought to be early events in the natural history of many cancers [1]. The second type of genetic change influence the cells' ability to migrate/move. Genes of the second type, while commonly associated with metastases, are also affected in primary tumors [9].

These two types of variation are thought to be implicated in malignant transformations for many (if not all) types of solid tumors. How do the two types of change trade-off to create a mutant which is "fitter" than the background? Questions of this kind are related to the general theory of fitness landscapes, first introduced by [10]. Fitness is viewed as a surface in a multidimentional space, where the dynamics is assumed to be directed toward local fitness maxima. The global maximum corresponds to the evolutionarily stable strategy [11]. In scenarios where fitness of an individual strategy depends on the current composition of the population (frequency-dependent fitness), the formalism of fitness generating functions is used [12].

In this paper we focus on a specific aspect of the general problem of fitness landscapes. Namely, we provide a qualitative framework to study the forces of selection acting within a spatially distributed, stochastic colony of cells, which can vary with regards to the two above mentioned characteristics. The models we construct for this purpose are a spatial generalization of a well-known Moran process, which was first introduced in [13]. This process has been used recently in cancer modeling (see [14-18]). The first spatial (1D) generalization of the Moran process was described in [19], where we considered the process of one-hit and two-hit mutant fixation. The simplicity of the (generalized) Moran process enabled us to study analytically, as well as numerically, the role of space in the processes of loss-of-function and gain-of-function mutations, see also [20]. In this spatial Moran process, the cells were allowed to divide in response to a death of a neighboring cell on a 1D grid.

In this paper we construct two spatial generalizations of the Moran process with cell migration. The first model includes explicitly the processes of cell division, death and migration. The second model implicitly describes migration through the varied ability of cell types to place their offspring a distance away, in response to another cell's death. The advantage of the simplified model is its analytical tractability. The two main findings are as follows:

• In both models, we find that migration has a direct positive impact on the ability of a single mutant cell to invade a pre-existing colony. A decreased fitness due to lesser growth potential may be offset by an increase in cell migration.

• The neutral ridges (the set of all types with the invasion probability equal to that of the host cells) remain invariant under the increase of system size (for large system sizes), thus making the invasion probability a universal characteristic of the cells selection status.

Our work is somewhat different from a large body of recent literature where spatial cancer dynamics is studied by means of cellular automata or agent-based modeling (see the recent reviews [21-29] and the references therein). Rather than adding on many biological processes and subtleties to our model, we focus on understanding how just two forces, proliferation and migration, trade-off to influence the overall fitness of cells.

## Results

### Explicit motility increases the invasion probability

**Figure 1:**
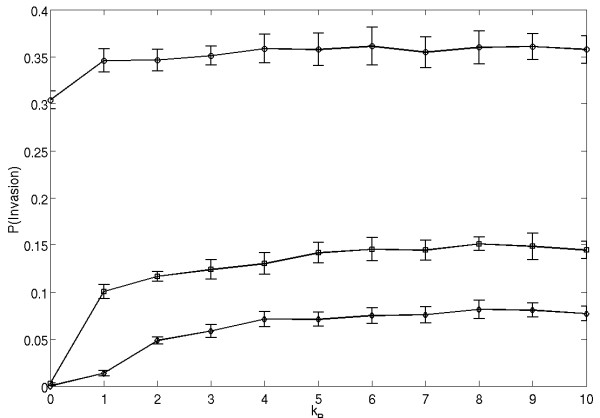
**Probability of invasion of mutant cells into a *k*_*A *_= 0 background with *λ *= 1.5 (circles), 1.0 (squares) or 0.9 (diamonds)**. Plots are the means ± SE of 10 sets of 10000 simulations.

We would like to investigate the effect of cellular motility on the probability of invasion of type B cells. We consider B-cells of higher (*λ *= 1.5), equal (*λ *= 1) and lower (*λ *= 0.9) division potential compared to that of the background cells A. We assume that the invading cells have a migration potential *k*_*B *_≥ 0, and that the background is nonmobile (*k*_*A *_= 0). We start from one cell of type B inserted randomly in the background of A cells, with the grid size of 21 × 21. 10 sets of 10000 simulations are performed, with each simulation running until 1 species is extinct. We then compute the probability of invasion for various migration rates. The results are plotted in figure 1 as the mean ± S.E. for each *λ*.

**Figure 2:**
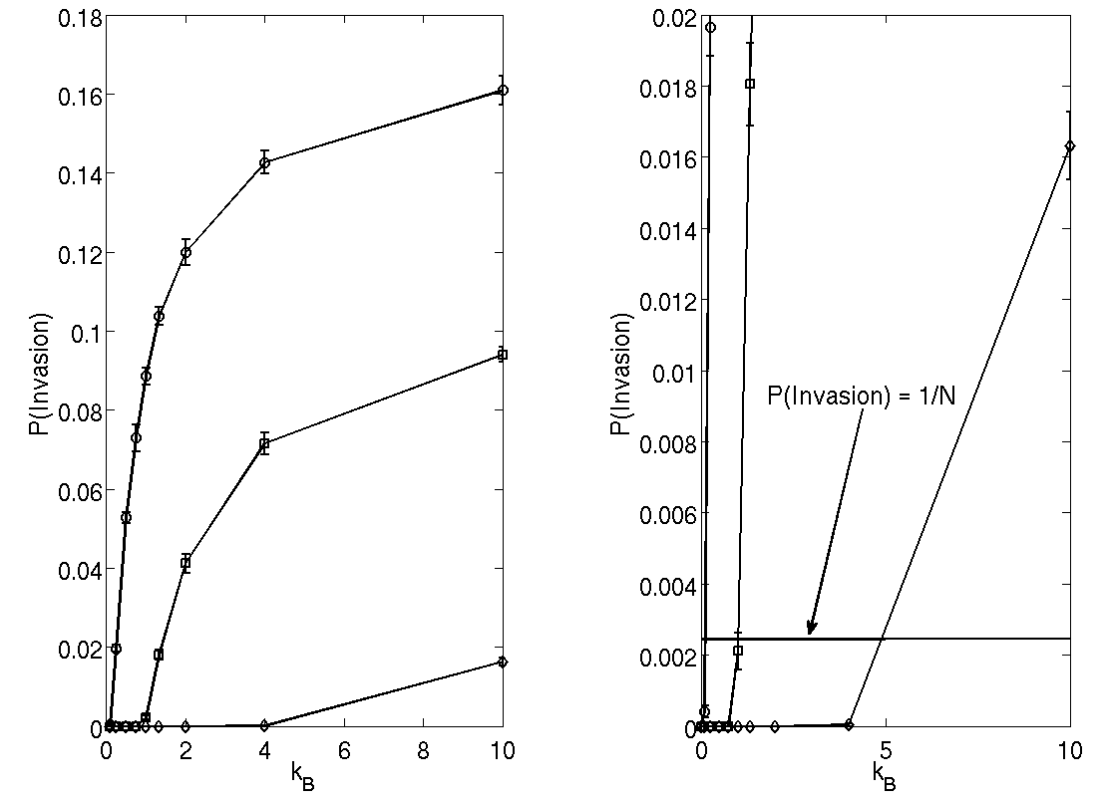
**(Left) Probability of invasion of mutant cells into a *k*_*A *_= 1.0 background with = 1.1 (circles), 1.0 (squares) or 0.9 (diamonds)**. Plots are the means ± SE of 10 sets of 10000 simulations. (Right) Close-up of the low probability regime of the plot. The line indicated corresponds to P(Invasion) = 1/N, the point which describes equal fitness between invader and background.

We discover that in this scenario motility has a positive effect on invasion probability. While the cells which were already dominant (circles) gained a slight extra advantage, even cells with lower division rates (diamonds) were able to invade when mobility rates became high. This effect is also present when the background cell type is itself motile as seen in figure 2 where *k*_*A *_= 1.0.

The result that an increased migration potential of mutant cells increases their ability to invade, can be explained intuitively. In the case of small mutant motility, mutant cells tend to concentrate in one region, and the expansion can only occur near the boundary of that region. An increase in mutants' motility increases the degree of mixing in the population, such that mutant cells spread throughout the space. In this case, mutant growth in enhanced as it can occur throughout the bulk of the colony.

### Identification of neutral ridges in the explicit model

Figure 2 shows that it is possible to find more than one pair of parameters (*k*_*B*_, *λ*) which correspond to the same probability of invasion. In other words, we can see that different strategies-that is, different parameter sets (*λ*, *k*_*B*_) relative to a specific background *k*_*A*_-may have the same invasion probability. It is particularly interesting to investigate these strategies for the probability of invasion  (see figure 2, right panel) which corresponds to the case where each cell as equal fitness (see Methods section below, as well as prior work, e.g. [15]).

**Figure 3:**
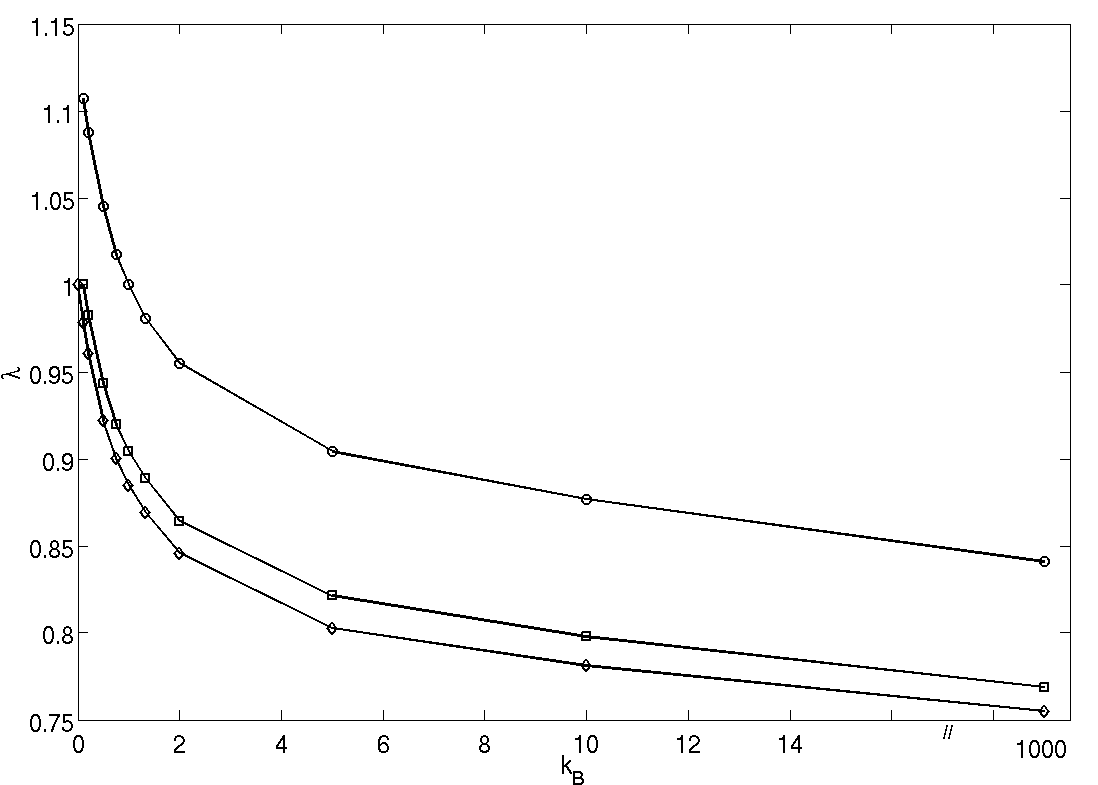
**Strategies of equal fitness (equal invasion probabilities) for background strategies of *k*_*A *_= 0 (diamonds), 0.l (squares) and 1.0 (circles)**.

In figure 3 we show sets of pairs (*k*_*B*_, *λ*) corresponding to *P *= 1/*N *for a fixed background, for three different values of *k*_*A*_. We observe that there is an inverse relationship between *λ *and *k*_*B*_. In other words, increasing the motility (*k*_*B*_) of invading cells will require a decrease in their reproductive potential (*λ*) in order to maintain the same level of invasion probability. An increase in either (or both) of parameters *k*_*B *_and *λ *leads to an increase in invasion probability.

We will say that all the strategies corresponding to the invasion probability *P *= 1/*N *are neutral to (have the same fitness as) the host cells A. We call the sets of all such strategies "neutral ridges". The relationship between the notions of invasion probability and fitness is discussed in more detail below. While we were unable to derive a simple law which relates the values of *k*_*B*_, *λ *and *k*_*A *_corresponding to *P *= 1/*N*, some features of their relationship are worth noting. Firstly, there appears to be an asymptote as *k*_*B *_→ ∞, implying for each background *k*_*A *_there is a minimum *λ *necessary for equal fitness strategies. That is, no amount of increased motility can compensate for too low a growth rate. Also, we notice that the distance between points on the *P *= 1/*N *lines remains approximately constant throughout the parameter regime (for example, the measured distance between the neutral ridges corresponding to *k*_*A *_= 1.0 (circles) and *k*_*A *_= 0.1 (squares), is constant for different values of *k*_*B *_with the accuracy of 2%). This implies that the law relating the strategy parameters *λ *and *k*_*B *_is somewhat independent of the background *k*_*A*_; the background parameter merely shifts the relationship between *λ *and *k*_*B *_vertically in the (*k*_*B*_, *λ*) plane.

### The role of the division radius in the implicit model

**Figure 4:**
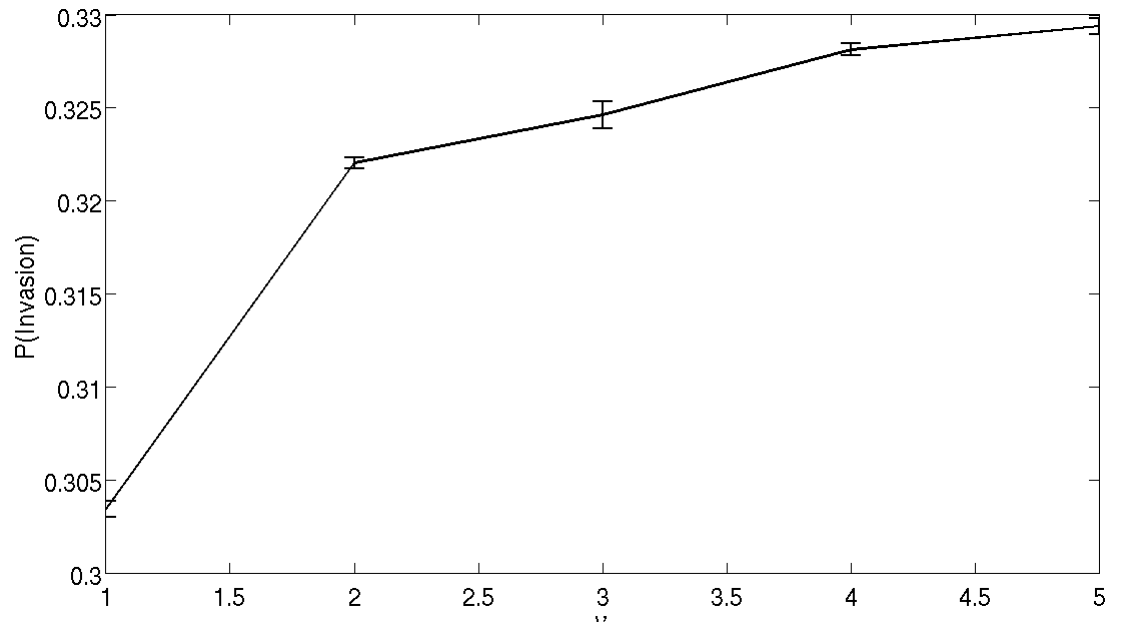
**The probability of mutant invasion (with *r*_*A *_= 1, *r*_*B *_= 1.5), as a function of cells' division radius, *ν*_*A *_= *ν*_*B *_= *ν***. The simulations are performed on a square grid of size 21 × 21.

We now begin analyzing the implicit model. To determine the effects that increasing the division radius has on the ability of a cell with superior replicative fitness to invade, a single mutant cell with replication potential *r*_*B *_= 1.5 is introduced into a background of cells with replication potential *r*_*A *_= 1, see figure 4. All cells (mutant and wild type) have division radius *ν*_*A *_= *ν*_*B *_= *ν*.

As seen in figure 4, increasing the division radius of all cells increases the ability of a superior mutant to invade the system. This is consistent with the previous result that in a spatial Moran process with *ν*_*A *_= *ν*_*B *_= 1, the probability of mutant invasion is smaller than that for a space-free model [19]. Increasing the division radius brings a spatial model closer to a space-free model. The space-free model is recovered as soon as the division radius is sufficiently big such that the entire domain is contained inside a circle of radius *ν*. This of course implies that there is a limit to the extent increasing the division radius can increase the invasion probability of a mutant phenotype. If the division radius already spans the entire space, increasing the radius further will have no effect on the probability of the mutant cell to divide. A cell with such a radius can be thought of as being governed by a nonspatial, bulk tumor growth model, such as a capacity growth or logistic law.

Interestingly, observe that increasing the radius from 1.0 (nearest neighbors) to 2.0 nearly recapitulates the theoretical results for a well-mixed system (invasion probability 0.33), see figure 4. This result is intriguing because, for a two dimensional grid of size 441 total cells (21 by 21), a division radius of 2.0 represents approximately 2.7% of the total area, and yet the results are nearly indistinguishable from a scenario in which all 441 cells can interact with each other.

### Level sets of invasion probability

**Figure 5:**
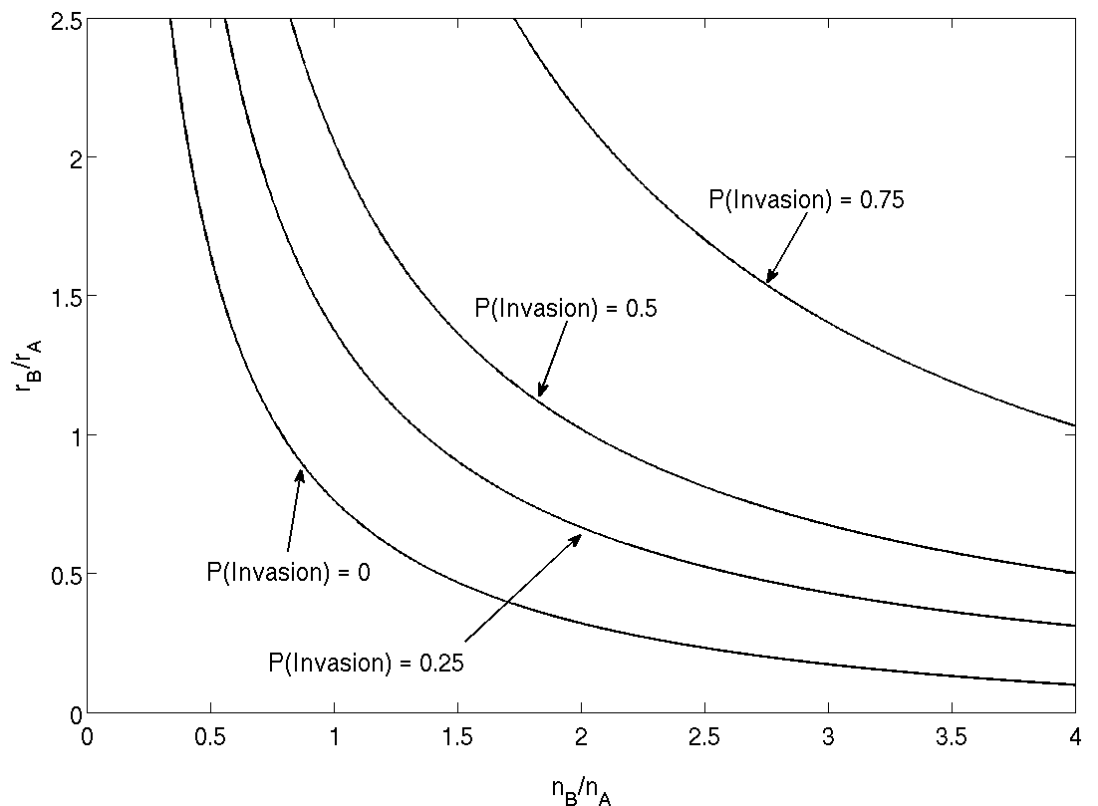
**The level sets of the mutant invasion probability in the parameters space (*n*_*B*_, *r*_*B*_)**. The background parameters are *r*_*A *_= 2 and *n*_*A *_= 20.

Let us explore how the changes in both the growth potential and division radius of a mutant influence its invasion probability. To that end, a single mutant cell with replication potential *r*_*B *_and division radius *ν*_*B *_is introduced into a background of cells with replication potential *r*_*A *_= 2.0 and division radius *ν*_*A *_= 2.5, which corresponds to *n*_*A *_= 20. Results are plotted in figure 5 as contour levels, or level sets, of invasion probability, as a function of *r*_*B *_and *n*_*B*_.

First of all, we observe that increasing *r*_*B *_and *ν*_*B *_(separately or together) leads to an increase in the invasion probability. This in itself is not surprising given that larger values of the growth potential and division radius will increase the mutants' probability to divide. Thus, if a cell is free to choose any values of *r*_*B *_and *ν*_*B*_, it will lead to an unrestricted growth of both of these parameters, which corresponds to the cells' climbing up the "hill" in the fitness landscape of figure 5, which corresponds to large *r*_*B *_and *ν*_*B*_. In reality though this is hardly possible and there are biological and energetic constraints on how often a cell divides, and how far it can travel upon division. Therefore "allowed" strategy trajectories in the (*r*_*B*_, *ν*_*B*_) space can be introduced where some external constraints do not allow a simultaneous large increase in both parameters. For example, if we look at trajectories of the type *α*_*r*_*r*_*B *_+ *α*_*ν*_*ν*_*B *_= *const*, where *α*_*r *_and *α*_ν _are some weights. We observe from figure 5 that the background condition of *r*_*A *_= 2.0 and *ν*_*A *_= 2.5 is able to resist most invasions from mutant strategies which maximize one trait at the cost of another (high *ν *with low *r *or vice versa). Thus, in this case the mixed strategy is more fit than those relying heavily on only one trait.

### Identification of neutral ridges for implicit motility model

We will use the parameters of figure 5 and proceed according to the following algorithm. By definition of the division radius, small changes in *ν*_*B *_may or may not lead to a change in the number of sites within division range. Therefore, we start by selecting a set of division radii *ν*_*B*_, and use the trace of the line of equal invasion probability to provide initial estimates for a value of *r*_*B *_for each *ν*_*B*_. We then perform simulations over a small range of *r*_*B *_centered at the initial estimate and proceed until we have found a candidate value *r*_*E *_for which the invasion probability  after 10 sets of 10000 simulations. We then confirm that the candidate pair (*r*_*E*_, *ν*_*E*_) is in fact a strategy of equal invasion probability by performing the reverse experiment; that is, introducing a mutant cell with strategy (2.0, 2.5) into a background population of strategy (*r*_*E*_, *ν*_*E*_).

**Figure 6:**
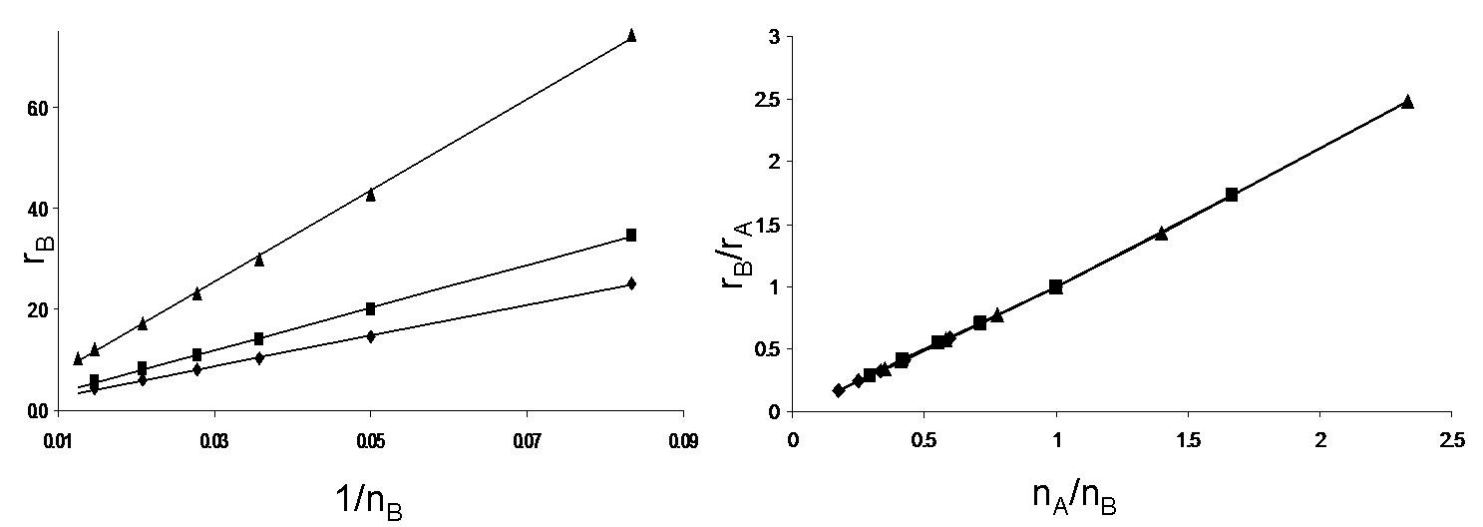
**Inverse relationship between *r*_*B *_and *n*_*B *_for strategies of equal fitness (*P*(Invasion) = 1/*N*)**. (Left) Strategies of equal fitness were computed for background strategies of (*r*_A_, *ν*_*A*_) = (2.5, 2.0) (diamonds), (2.0, 2.5) (squares), and (3.0, 3.0) (triangles). (Right) Plot of the same strategies of equal fitness scaled to their background environment.

The results of this and similar experiments with other choices for *r*_*A *_and *ν*_*A *_are presented in figure 6. In this context, it is more convenient to talk about the number of neighbors, *n*, for each type, rather than about their division radii, *ν*. By the number of neighbors we mean the number of slots in the vicinity of the cell which are within the division radius *ν *of the cell. In figure 6, the mutant growth potential, *r*_*B*_, is plotted against the inverse of number of neighbors,  (see left panel). The three lines correspond to three different background conditions, including the one corresponding to figure 5 (squares). The numerically calculated values are plotted together with the fit by the function(1)

In each case, the correlation coefficient is greater than 0.99, which confirms the observation that the growth potential and the division radius of the cells of constant invasion probability are inversely proportional to each other.

The right panel of figure 6 replots the same data in terms of the relative growth potential, *r*_*B*_/*r*_*A*_, and the relative number of neighbors, *n*_*A*_/*n*_*B*_. We observe that the three neutral ridges from the left panel align.

We deduce that the neutral ridges satisfy the relationship *r*_*A*_*n*_*A *_= *r*_*B*_*n*_*B*_.

In order to understand these results, let us consider the following process. At each time-step, we ask: what is the probability that a given cell, *i*, will divide in the next time-step? This is proportional to the probability that an empty space appears within radius *ν *of the given cell, which is given by *n*_*i*_/*N*, and is weighted with each cell's growth potential:

Once this quantity is calculated, we "flip a coin", and replace an arbitrarily chosen cell with a cell of the given type. In this process, in order for the cells of type *B *to have the same invasion probability as cells of type *A*, the probability of cell division for cells of type A should be equal to the probability of division of cells of type B. That is, *r*_*A*_*n*_*A *_= *r*_*B*_*n*_*B*_. Resolving this equation, we obtain *n*_*B *_= (*n*_*A*_*r*_*A*_)/*r*_*B*_. This is an inverse dependence with the proportionality coefficient *c *= *n*_*A*_*r*_*A*_.

### Invasion probability and fitness

So far we have been investigating the probability of invasion of type B starting from one such mutant. This probability is connected to the *relative fitness *of type B, which we denote as ℱ. Relative fitness is a parameter that is related to the rate of expansion of a phenotype. It is usually defined as the (averaged) frequency of the type in the "next" generation divided by that in the current generation: . A related concept of *invasion fitness *is defined as the exponential growth rate of the mutant type in a host population. The connection of the relative fitness parameter (ℱ) to the probability of invasion (*ρ*) has the following useful properties:

(i) If *ρ *= 1/*N *(neutral mutant B) then ℱ = 1 (the number of neutral cells of type B stays constant on average, from generation to generation).

(ii) If *ℱ *→ ∞, that is, if the mutants are strongly advantageous, then *ρ *→ 1.

(iii) The invasion probability and fitness are positively correlated (the types that have a higher invasion probability *ρ *will have a higher fitness and vise versa).

The notions of fitness and probability of invasion are both important in theoretical biology, and both have advantages and disadvantages. Arguably, the notion of invasion probability is more informative in our setting.

The fitness parameter is defined for a particular temporal dynamics. In our simple model, we use a discrete-time Markov chain. This is an unrealistic way to treat the mutant dynamics, but it preserves the invasion probabilities. In other words, if one considers the long-term outcomes of a stochastic process, the dynamics becomes unimportant, and can be simplified in the way implemented here. The expansion rate of cells (equivalent to fitness) is dependent on this simplification and thus is affected by this (unrealistic) aspect of our model.

The probability of invasion does not depend on the exact way we choose the time-step for our updates. Thus in this sense it is a better choice of a competitiveness measure for our system. On the other hand, invasion probability depends on the total number of cells, *N*. The probability to invade a very small constant-size population is higher than that for a large colony. We have investigated how the probability of invasion depends on *N*, and found the following.

For non-neutral mutants, probability of invasion is a monotonically decreasing function of *N *which saturates for large values of *N*. In our experiments, we used 2D grid size 21 × 21. We have also experimented with the size 31 × 31 (which yields *N *more than twice the original size), and found no measurable change in the results (not shown). More precisely, the calculated values of the mean invasion probability for the larger grid size were within the standard deviation of the mean obtained by the smaller grid size, and vice versa. This is similar in spirit to our earlier analytical results for the invasion probability of the space-free Moran process (see equations (2)), and a 1D Moran process without motility (equation (3)). In that case, as long as |*r *- 1|*N *≫ 1,

A more subtle situation arises when the mutant B is neutral. Again using the example of our earlier analytical findings, we can see that if |*r *- 1|*N *≫ 1,

that is, the invasion probability strongly depends on *N*. Despite this fact, the notion of invasion probability retains a degree of universality even in the case of neutral mutations. Namely, we found numerically that the expressions for the neutral ridges given by functions *r*_*B*_*n*_*B *_= *c *are *N*-independent, that is, the proportionality constant *c *does not change with *N *(not shown). This is consistent with the derivation of the parabolic dependence of the level sets for the invasion probability.

Ultimately, we are interested in the probability of invasion as long as it is equivalent to the probability of mutants to thrive. When considering cancer, it is irrelevant whether exactly all the host cells in an organ have been replaced by the mutants (which is equivalent to invasion, rigorously speaking). An important notion is whether a mutant colony expands and persists inside an organ for a long time, which, for large values of *N *and for advantageous mutants, is very close to the probability of invasion. This notion is more universal than the fitness of mutant cells because it is independent of the time-evolution or the system size. Another meaningful concept is neutrality, which is basically a symmetry property: a neutral mutant behaves just like any host cell, and has the same invasion probability as any other cell (1/*N*). Its fitness is 1 and its expansion rate (invasion fitness) is zero, which is extremely hard to measure. Instead, measuring the invasion probability gives us a useful tool to identify the class of neutral mutants.

In the rest of this section we will discuss the probability of invasion and make some inferences about the fitness of the mutants. As explained above, the two notions are positively correlated, so all the arguments resulting from considering the level sets of the invasion probability hold for the mutant fitness.

### The optimal mutant strategy

**Figure 7:**
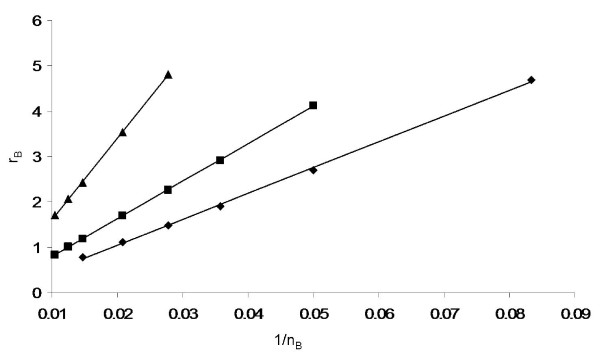
**Inverse relationship between *r*_*B *_and *n*_*B *_for strategies of fixed invasion probability**. Strategies with invasion probability 0.25 (diamonds), 0.5 (squares) and 0.75 (diamonds) were computed for the background strategy in figure 5, ((*r*_*A*_, *ν*_*A*_) = (2.5, 2.0).

We have established so far that the mutant cells of the fitness equal to that of the host satisfy the relation *r*_*E *_= *c/n*_*E*_. This type of relation applies to other constant levels of invasion probability. In figure 7 we present the numerically obtained inverse relationship between *r*_*B *_and *n*_*B *_for strategies of fixed invasion probability; these lines correspond to the level sets in figure 5. The numerically calculated points are plotted together with the hyperbolas (which look like straight lines in the coordinates we use here); as in figure 6, the correlation coefficients are greater than 0.99. We can see that for cells of a given invasion probability we have *r *∝ 1/*n*, which means that the fitness function *ρ *(defined as the probability of type B to invade) satisfies

Its levels correspond to a family of hyperbolas *r*_*b*_*n*_*B *_= *c*. Using the arguments of the previous section, we deduce that the same holds for the fitness function:

Moreover, we know that the function ℱ is a monotonically increasing function of its argument, because the fitness increases both with the growth potential and the division radius. Thus we have

Using this information we can make some progress in identifying likely evolutionary strategies of mutants which aim to maximize the function ℱ. Let us suppose in all generality that external biological and energetic constraints impose a relationship between the allowed values of *r*_*B *_and *n*_*B*_, which we can write in the form

We further assume that this equation can be solved for *r*_*B *_to obtain

and thus the characteristic levels of the function ℱ are given by

We can also safely assume that *g' *≤ 0, because of the nature of the constraints we consider; simply speaking, an increase in the growth potential must lead to a somewhat reduced division radius, thus making the function *g *monotonically increasing. The function *n*_*B*_*g*(*n*_*B*_) can take a variety of shapes. Here we restrict ourselves to the cases where it has at most one internal extremum. Multiple minima and maxima could occur, but they are a result of specific biological facts which we cannot specify at the present level of generality. Thus we focus on the simplest types of generic behavior, keeping in mind that other functions can be studied in a similar way. With at most one internal extremum, there can be the following cases:

• The function *n*_*B*_*g*(*n*_*B*_) has an internal maximum. Then the function ℱ also has an internal maximum at the corresponding point (*ν*_*B*_, *r*_*B*_). This means that the optimal strategy is to find an intermediate value of growth potential and division radius, instead of maximizing them both.

• The function *n*_*B*_*g*(*n*_*B*_) is monotonically decreasing; this could be a result of a constraint that small values of *ν *are not allowed. Then, the maximum of *F *is achieved for smallest possible values of *n*_*B *_and the largest possible values of *r*_*B*_. In other words, the optimal strategy is maximizing the growth potential (within the allowed range) at the cost of the division radius. This situation could arise in the following scenario: suppose that increasing *ν *is "expensive", that is, the constraint function *f *(*r*_*B*_, *ν*_*B*_) depends stronger on *ν*_*B *_than it does on *r*_*B*_. Then, the value of the derivative, |*g'*|, is relatively large, which shifts the location of the maximum of the function *ν*_*B*_*g*(*ν*_*B*_) to the left. If the dependence of *ν*_*B *_is sufficiently strong, then this can drive the location of the maximum outside the allowed domain of *ν*_*B*_. As a result, the optimal strategy will be to find the smallest possible *ν*_*B*_.

• The function *n*_*B*_*g*(*n*_*B*_) is monotonically increasing in the allowed domain. Then, the maximum of *F *is achieved for largest possible values of *n*_*B *_and the smallest possible values of *r*_*B*_. Analogous to the previous argument, such scenarios could arise when increasing *ν*_*B *_is "cheaper" than increasing *r*.

To summarize, we find that the growth potential and the division radius are two components of fitness which play a very similar role in the probability of mutant invasion. The fitness landscape has hyperbolic level sets in terms of the growth potential and the number of neighbors. A mixed strategy is optimal unless one of the two fitness components (the growth potential or the number of neighbors) is much more evolutionary "expensive". In the latter case the less expensive characteristic should be maximized at the expense of the other.

## Discussion

A central idea of this paper is that the cell with the fastest intrinsic growth rate is not always the most fit. Rather, it is the culmination of *ability *to divide with increased *opportunity *to do so. These forces must be balanced, depending on the relative costs of both adaptations for a cell.

We have demonstrated how relatively simple, two-component, models can help explore the complicated phenomenon of phenotypic heterogeneity. With the multiphasic nature of fitness presented here, many different strategies have equal or nearly equal fitness (figures 3 and 6). As the number of traits under consideration increases, it is similarly expected that the potential combinations of strategies which lead to equal fitness will increase. This would reflected in the existence of multidimentional sets equivalent to neutral ridges described here.

The heterogeneous nature of tumors provides a primary mechanism for resistance to current therapeutic strategies. Tumors behave as fast evolutionary systems, with many subtly different phenotypes coexisting, cooperating and/or competing within a shared environment. Changes in phenotypes can arise de novo from altered gene expression profiles, rapid cell proliferation outpacing DNA repair mechanisms, loss of repair systems or checkpoints, alterations to chromosomal integrity and epigenetic events. The application of therapy (chemical, radiation) to this system changes the selective forces acting upon the various phenotypes, permitting rigorous selection and so-called "punctuated evolution" within the tumor [30]. These external forces can act to select and advantage a phenotype that might otherwise be unlikely to achieve dominance, or to accelerate the rise of a given phenotype within a population.

### Fitness ridges, fitness landscapes and chemotherapy

Assessing the effectiveness of a therapeutic intervention, via mass die-off and selection, underlies the difficulty in studying, understanding and treating cancer. The plasticity of the disease will alter parameters as the disease is treated. Clearly, a predictive understanding of how mutants penetrate both pre-treatment and post-treatment tumors, will be critical for designing new therapy strategies.

Our model can help understand how therapy influences the fitness landscape of the tumor. We test the hypothesis that a higher death rate generates a positive selective pressure on a more mobile phenotype. Such a death rate might describe a form of medical intervention. To test this hypothesis, we allow significantly more than one cell to die per iteration of our process. We begin with a system composed of 10% mutant cells in a background of wild type. At each step of the iteration, each cell in the space has an independent probability to die of *P*_*d*_. Thus, after the mass death there will be on average (1 - *P*_*d*_) * *N *cells remaining in the system. The process described previously is then simulated without any new cell death until the space is filled again. At the end of each such iteration we determine the number of type A and type B cells in the system. This process is repeated for a fixed number of mass death events. We also determine whether or not one cell type has completely invaded the system.

**Figure 8:**
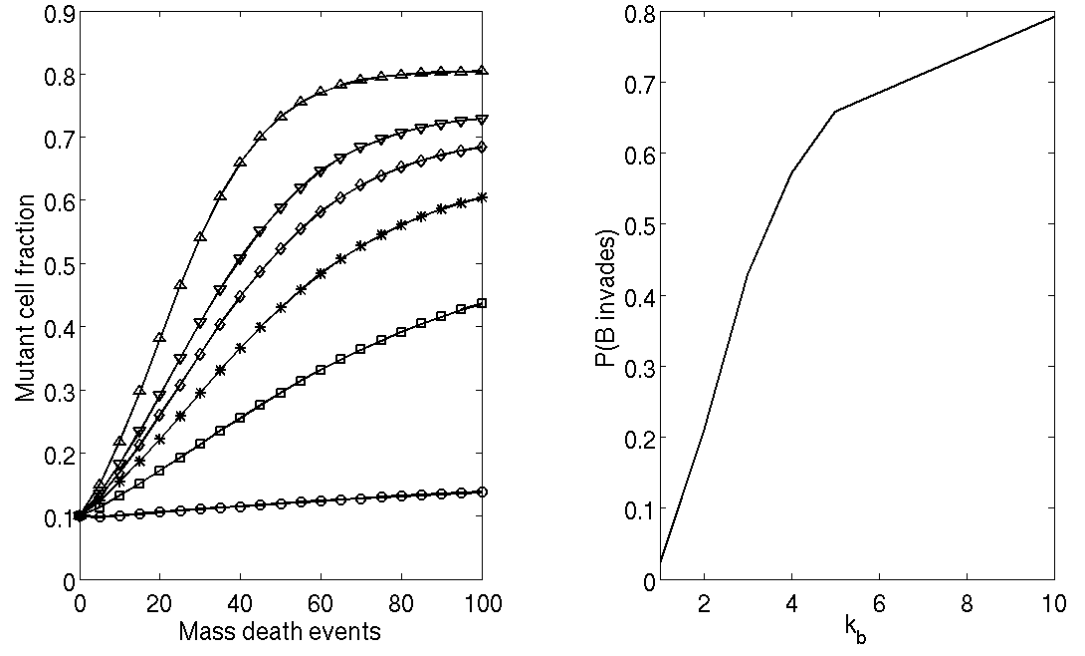
**Effects of a mass death probability on motility phenotype fitness**. (Left) Enrichment of slower growth, higher motility mutant cells (*λ *= 0.8, *k*_*B *_> 0) into a background of *k*_*A *_= 0 cells under successive iterations of a mass death. *P*_*d *_= 0.8. Plots correspond to *k*_*B *_= 1 (circles), *k*_*B *_= 2 (squares), *k*_*B *_= 3 (asterisks), *k*_*B *_= 4 (diamonds), *k*_*B *_= 5 (downward triangles), and *k*_*B *_= 10 (upward triangles). (Right) Probability of invasion of *λ *= 0.8 mutant cells as a function of motility strength (*k*_*B*_) under a mass death assumption (*P*_*d *_= 0.8).

As seen in figure 8, increasing the number of cells which die at each iteration strongly increases the ability of the mutant cell to invade the system. In this figure, we have chosen a background phenotype of *k*_*A *_= 0 and an invading phenotype of *λ *= 0.8, *k*_*B *_as given. Note that as shown in figure 1, this mutant is a poor invader in the single death model; a mutant with a higher replicative fitness (*λ *= 0.9, diamonds) was able to invade the background less than 10% of the time at maximum motility. We contrast this result to figure 8, which shows that the increased death rate clearly favors the more mobile phenotype. We observe enrichment of mutant cells as iterations progress for all values of motility strength (*k*_*B*_) (left panel). Furthermore, there is a striking increase in the probability of mutant invasion (right panel).

Thus, we have demonstrated that application of a toxic therapeutic intervention that results in gross tumor death can significantly promote the invasion of a more motile mutant into the general tumor- in fact, our data suggest that this advantages the invasive capacity of a motile tumor relative to the faster proliferating tumor. This somewhat unexpected observation may reflect the clinical situation; many malignant tumors exhibit enhanced expression of genes that promote motility, as discussed below.

In the implicit migration model considered here we found that the the fitness of cells depends on two parameters, the growth potential and division radius. Moreover, we demonstrated that it depends on the product of the two. This is not an unusual finding in evolutionary biology. Another example comes from virus dynamics, where the fitness of viruses (measured in terms of their basic reproductive ratio) can be calculated as a function of various parameters in predator-prey type models. It is typically proportional to the product of two quantities: the infectivity of the virus and the inverse of the death rate of infected cells. The former parameter can be roughly related to the "motility" of the virus, or to how efficiently it infects new cells, and in this sense it is roughly analogous to our division radius. The inverse death rate of infected cells is reflective of our growth potential since a decrease in the cell's lifespan (a decrease in the inverse death rate) leads to a decrease in the total number of progeny.

Several papers have been devoted to studying optimal viral strategies, especially given that the two components of fitness are not independent [31-38]. In spirit, our approach is similar to that. While there has to date been little investigation into this area, it seems reasonable to assume energy availability limits the total growth and migration ability of an invasive cancer cell; that is, since both growth and migration are energy-intensive processes, an invasive cell will have to 'budget' its energy to each.

### Biological Applications

The model presented in this paper is a very crude approximation of reality. Thus the prediction generated by the model may or may not be relevant for biological applications. However, it is particularly compelling that several genetic alterations have been discovered which are implicated in promoting migratory and/or invasive phenotypes, yet play no direct role in regulating cell division. The in vivo selection for these phenotypes would appear to indicate a strong selection for the fitness conferred. For example, genes that regulate cell interaction with the local extracellular milieu, [39] or those that promote cytoskeleton dynamics [40] have been established to promote cell migration.

The class of small GTPases has been generally divided into families, based on homology and role played in the cell. The Ras family members k-ras, n-ras and h-ras have been linked to cell proliferation, while Rho family members such as Rac, RhoA and RhoC and Rac are small GTP binding proteins the influence cytoskeletal remodeling [41]. These diffierent family members exhibit significant cross-talk, with Ras influencing motility somewhat and Rho proteins sometimes contributing to proliferation. The capacity of numerous cellular proteins found to be elevated in cancer to influence both proliferation and motility, and include a swath of oncogenic proteins such as Src family kinases [42] mitogen activated family kinases [43], and phosphoinnositide 3 kinases [44].

Nonetheless, cytoskeletal effectors which are tightly linked to migration do promote cell invasion and tumor malignancy. Effectors such as RhoC are not oncogenes per se, as they do not transform primary cells, but nonetheless influence cell migration and tumorigeneicity [45,46]. Moreover, other mediators in the cytoskeleton control system have been shown to impact migration potential and tumorigenicity. The protein ABI-1 is an adaptor molecule which, upon RhoA activation, facilitates the formation of multiprotein complexes involved in lamellipodia formation [47]. Overexpression of ABI-1 correlates well with migratory and invasion potential in breast cancer cell lines, while suppression of this protein led to the complete loss of migratory ability in once highly invasive cell lines [47].

Coupled to the activity of the cytoskeleton are the cell surface receptors mediating extracellular matrix interaction. Integrins, and their associated proteins, have been shown to be critical for motility and survival. For example, the integrin associated protein focal adhesion kinase (FAK) is a critical determinant of cell migration, yet similar to the small GTPases of the Rho family, the expression of FAK is insufficient to transform primary cells [42,48]. Moreover, FAK expression is not essential for tumorigenesis, yet FAK expression promotes tumorigenicity and invasion. Accordingly, FAK is found to be enriched in aggressive human tumors. The common theme emerging from these data is that, increased migratory capacity results in a tumor cell with increased fitness. It is not yet clear whether this fitness is sufficient to result in a phenotypic conversion of a tumor in vivo, and it would therefore be interesting to determine whether this occurs in a preclinical model of tumor development. However, our simplified model provides very similar results to those seen in vivo with respect to the importance of migration, and provides an interesting perspective on tumor cell fitness during tumor progression. While the opportunity to divide may often be related to factors that govern the intrinsic proliferative potential of the individual cells, our data suggest that migration can compensate for a lower proliferative rate, and that migration may therefore be an important selection factor during tumor progression in patients. Strikingly, the study also points out the potential for chemotherapy to influence tumor phenotype in an unintended manner.

## Conclusions

We have formulated two spatial extensions of the Moran process to study the effects of motility on the ability of a new mutant cell to invade a pre-existing tumor. Our results indicate that a mobility phenotype can be dominant and able to invade a background of cells with higher reproduction rates. Further, we have investigated how differing combinations of replication and motility strength blend to form equivalent fitnesses within the tumor system. Finally, we have shown how outside influence on the system inducing mass death within the tumor might unintentionally favor invasion and regrowth by a more aggressively motility phenotype.

## Methods

### Previous work: the Moran process

The conventional Moran process is formulated as follows. In a population of *N *cells, each cell is equipped with a nonnegative replication rate. The process is a sequence of updates. At each time-step, one cell is chosen randomly for death and is then replaced by a progeny of another cell (note that in this process, the probability to be picked for death is the same for all cells, regardless of their type). To choose which cell reproduces, one weighs in all cells replication parameters, such that the probability for cell *i *to reproduce is given by , where *r*_*k *_is the replication rate of cell *k*, and the summation is performed over all cells in the population.

In we assume that there are two distinct types in the population, type A with replication parameter *r*_*A *_and type B with replication parameter *r*_*B*_, then the stochastic process can be formulated in terms of only one independent random variable, the number of cells of type B. If there are *i *cells of type B, then after one update the following transitions are possible:

• *i *→ *i *+ 1 with probability *P*_*i *→ *i*+1 _= (*N *- *i*)/*N *× *r*_*B*_*i/*(*r*_*A*_(*N *- *i*) + *r*_*B*_*i*), where the first term is the probability that a cell of type A dies and the second term is the probability for a cell of type B to divide;

• *i *→ *i *- 1 with probability *P*_*i *→ *i*-1 _= *i*/*N *× *r*_*A*_(*N *- *i*)/(*r*_*A*_(*N *- *i*) + *r*_*B*_*i*), where the first term is the probability that a cell of type B dies and the second term is the probability for a cell of type A to divide;

• *i *→ *i *with probability 1 - *P*_*i *→ *i*+1 _- *P*_*i *→ *i*-1_.

We will refer to cells of type A as the host, or background, cells, and cells of type B as invading cells. In the Moran process as it is formulated (that is, in the absence of new mutations), only two outcomes are possible: either type A wins and cells of type B disappear, or type B wins and cells of type A disappear. The probability for mutants to invade starting from one cell can be calculated analytically and is given by(2)

In the special case of neutral mutants, *r*_*A *_= *r*_*B*_, we have *ρ *= 1/*N*. This can be obtained from formula (2) by taking the limit *r*_*B *_→ *r*_*A*_. Also, this result follows from symmetry considerations: a cell of type B if type B is neutral has the same expansion properties as any of the host cells, and the same probability to invade. Since inevitable one of the *N *cells will invade, the probability of invasion is 1/*N *for every cell, including the B cell.

### First spatial generalization of the Moran process

In [19] we introduced and analyzed a first spatial generalization of the Moran process. We considered a 1D space, where all the *N *cells were placed on a regular grid, at locations 1, 2,, *N*. As before, we assume that the total number of cells does not change. Cells are randomly chosen for death. Each cell death is followed by a cell division of one of its two neighboring cells, which places its daughter cell at the empty slot. Cell death occurs randomly and division is proportional to the relative fitness of the cells.

In this spatial model, the probability of mutant invasion in principle depends on the initial position of the mutant cell. However, if we use periodic boundary condition, this dependence disappears and we obtain that starting from one cell, the probability of invasion is(3)

where *r *≡ *r*_*B*_/*r*_*A*_. It can be shown that  ≤ *ρ*, with the equality corresponding to neutral mutants, *r *= 1.

In other words, for nonneutral mutants, the probability to invade is smaller in a spatial model than it is in the space-free Moran process.

Note that both the space-free Moran process and the spatial generalization described above depend only on the ratio of the replication parameters *r*_*B*_/*r*_*A*_.

### The explicit motility model

Our aim in this paper is to study selection processes acting upon cells in spatial settings. The first step toward that goal was made by creating a straightforward spatial generalization of the Moran process described above. However, this model is too simplistic. For example, starting from one mutant cell, a mutant colony can only spread as a solid, connected spot. This is a consequence of the facts that (i) the model is one-dimensional, (ii) cells only interact with their nearest neighbors and (iii) cells are not allowed to migrate.

In this paper we introduce two spatial modifications of the Moran process which are higher-dimensional, and include cellular motility. This section describes a model in which cell motility is explicitly incorporated. A simpler model, in which motility implicitly described, is discussed in the next section.

#### Spatial Moran algorithm

Suppose that *N *cells are placed on a rectangular grid in 2D (the algorithm generalizes straightforwardly to 3D), where there is a cell of type A or B at each node. As in the standard Moran process, the first event of an iteration is the random selection of one cell to die. Then, one of four events are possible:

1. a background cell divides;

2. an invading cell divides;

3. a background cell migrates;

4. an invading cell migrates.

In all these cases, the empty spot created by the initial cell death is filled. If a migration event occurred, a new empty space is created. The division and migration events occur with rates *r*_*X *_and *m*_*X *_for *X *= *A, B *respectively, and are contingent upon the number of each type, *A *and *B*, within nearest neighbors distance of the empty space. If a division event occurs, the iteration step is complete and a new cell is selected for death. If a migration event occurs, the migrating cell 'trades places' with the empty space and a new event (division or migration) is selected. Thus, at the beginning and end of each iteration, the environment is completely filled; however, there may be many migration steps before a division event ends the step. If *n*_*A *_and *n*_*B *_are the number of type A and B cells within the nearest neighbor range of the empty site, then the probabilities of each event are:

where we denoted *K *= *n*_*A*_(*r*_*A *_+ *m*_*A*_) + *n*_*B *_(*r*_*B *_+ *m*_*B*_).

In our simulations, we use a square grid with periodic boundary conditions. At time 0 we introduce a single type B mutant at a random position in the space grid which is otherwise filled with type A cells. The process described above is repeated until one species, A or B, has been eliminated from the environment.

#### Model parameters: the growth and migration potentials

The explicit motility model is defined in terms of four cellular characteristics: the growth potential, *r*, and the migration potential, *m*, of the two cell types. We can normalize the system to reduce the number of parameters to three. Dividing the numerators and the denominators of various probabilities by *r*_*A *_yields:

where  is the ratio of migration over division potentials,  is the ratio of invader to background growth potentials, and  = *n*_*A*_(1 + *k*_*A*_) + *n*_*B*_*λ *(1 + *k*_*B*_).

Note that increasing the migration potential in this model is similar to decreasing the typical time-scale of migration compared to that of cell division. It amounts to an increase in the average distance traveled by a cell in each iteration. An assumption of the model is that the death rate of cells is low compared to the division rate. In other words, we typically do not expect to have multiple cell deaths within a migration radius of a given cell. This is why we can complete each iteration (from a cell death event to a division event) independently.

### The implicit motility model

The advantage of the model described in the previous section is the explicit representation of motility within the cell population. A disadvantage is that the relationships for determining equality amongst strategies were found to be too complex to study analytically. Thus, we designed a simpler model of motility, in which cell movement is implicitly tied to the survival of new cell progeny. In this section we present the formulation and the analysis of this simpler model.

#### Model Scheme

Following the standard Moran process, at each time-step, one cell is selected randomly to die, to be immediately replaced by the progeny of one of its neighboring cells. As before, the difference between the two phenotypes A and B is reflected in their ability to reproduce. Suppose that cells of type A have growth potential *r*_*A *_and division radius *ν*_*A*_, and cells of type B have growth potential *r*_*B *_and division radius *ν*_*B *_(the distance over which a cell can place its progeny); the biological meaning of these parameters is discussed in the next subsection. The probability for the empty slot to be filled by either the background (A) type or mutant (B) type depends on the division radii and growth potential of each.

Let the discrete parameters *s*, *s' *vary over all the points in the grid, and denote *d*(*s, s'*) the Euclidean distance between the points *s *and *s'*. Further, we use indicator function *A*_*s *_(*B*_*s*_) to take the value of 1 if point *s *contains a cell of type A (B), and zero otherwise. Then, the probability that a given empty slot *s *is filled by a progeny of a cell of type A is given by(4)

The probability for the slot to be filled by a progeny of a cell of type A is *P*_*B *_= 1 - *P*_*A*_.

Note that the first spatial Moran process described above is a special case of the implicit model which corresponds to a 1D space and *ν*_*A *_= *ν*_*B *_= 1.

#### Model parameters: the growth potential and the division radius

In the process described above, each cell is characterized by two parameters, *r*_*A *_(or *r*_*B*_) and *ν*_*A *_(or *ν*_*B*_). We call the former the *growth potential *and the latter *division radius*. Growth potential is a measure of, given that an empty space has opened sufficiently close to a cell, how likely it is that that cell will divide and fill the space. The corresponding parameter, *r*, can take any positive values. It is not in any sense a probability to divide, it instead measures how often the cell can divide compared to other phenotypes in the colony. Thus, the results will only depend on the ratios of growth potentials.

The division radius is a measure of what "sufficiently close" in the prior definition represents. From a technical standpoint, a division radius of 1.0 in a 2D square grid corresponds to nearest neighbor interactions, while a radius of 1.5 corresponds to next nearest neighbors.

This implicit model only tracks *net *division and migration events. Further, the model depends only on two parameters: the relative growth potential *r*_*B*_/*r*_*A *_and the relative division radius *ν*_*B*_/*ν*_*A *_(this follows from equation (4). Thus, it is a simpler to analyze than the previous model that accounts for migration explicitly. Note that as currently implemented, all cells within the division radius have equal growth potential. It is straightforward to generalize the model such that the growth potential depends on the distance from the empty space.

It is important to note here that the implicit motility model is not intended to be a mechanistic model of growth and evolution of a spatially defined tumor. It is instead an abstraction of a more mechanistic system, the explicit motility model, made under a simplifying assumption, which allows a more analytic treatment of the results. The fundamental assumption allowing us to construct the implicit motility model is that the timescale of migration is much faster than the timescale of tumor cell death in a fully formed tumor. Thus, the division radius might be considered to be the average distance a daughter cell migrates away from a parent cell before the next death event occurs.

## Competing interests

The authors declare that they have no competing interests.

## Authors' contributions

CT assisted in formulating the models, performed the simulations, assisted with the analysis, and drafted the manuscript. JL assisted in formulating the explicit model and associated experiments. DS conceived of the mass death experiments and helped draft the biological applications for the manuscript. NK formulated the models, performed the analysis and helped draft the manuscript. All authors read and approved the final manuscript.

## Reviewers' Comments

**Glenn Webb**: There is a continuum modeling approach related to the issues raised in this paper given in the paper. Transforming growth factor TGF-beta is known to inhibit cell proliferation, and also increase cell motility and decrease cell-cell adhesion [49]. A version of the Fisher-Kolmogorov equation was used to quantify the simultaneous effects of TGF-beta to increase the tendency of individual cells and cell clusters to move randomly and to decrease overall population growth. Accompanying experiments demonstrated that TGF-beta increased the percentage of mobile cells in an in vitro cell population in a dose-dependent manner, consistent with model simulations. Have the authors tried to develop continuum models to quantify migratory impact on invasive cell population dynamics?

**Marek Kimmel**: The paper introduces very interesting spatial extensions of the Moran model with resulting conclusions concerning invasion by a mutant depending on cell motility and division radius. However, mathematical and simulation results provided, concern only aggregate characteristics such as invasion probability, without discussing the spatial patterns emerging. These latter might be interesting as demonstrated by a class of spatial models, which explore the consequences of linking the model of spatial growth of precancerous cells with diffusion of the growth factors [50]. The picture emerging from modeling indicates that production of growth factors by cells may lead to diffusion-driven instability, which in turn may lead either to decay of both population, or to emergence of local growth foci represented by spike-like solutions. Interesting dependencies arise when two mutualistic cell populations are considered. It would be probably interesting to check if any of the models in the current paper may lead to similar dynamics?
